# Chain-like structure elements in Ni_40_Ta_60_ metallic glasses observed by scanning tunneling microscopy

**DOI:** 10.1038/srep13143

**Published:** 2015-08-13

**Authors:** Rémy Pawlak, Laurent Marot, Ali Sadeghi, Shigeki Kawai, Thilo Glatzel, Peter Reimann, Stefan Goedecker, Hans-Joachim Güntherodt, Ernst Meyer

**Affiliations:** 1 Department of Physics, University of Basel, Klingelbergstr. 82, 4056 Basel, Switzerland; 2 Department of Physics, Shahid Beheshti University, Evin, 19839 Theran, Iran

## Abstract

The structure of metallic glasses is a long-standing question because the lack of long-range order makes diffraction based techniques difficult to be applied. Here, we used scanning tunneling microscopy with large tunneling resistance of 6 GΩ at low temperature in order to minimize forces between probe and sample and reduce thermal fluctuations of metastable structures. Under these extremely gentle conditions, atomic structures of Ni_40_Ta_60_ metallic glasses are revealed with unprecedented lateral resolution. In agreement with previous models and experiments, icosahedral-like clusters are observed. The clusters show a high degree of mobility, which explains the need of low temperatures for stable imaging. In addition to icosahedrons, chain-like structures are resolved and comparative density functional theory (DFT) calculations confirm that these structures are meta-stable. The co-existence of icosahedral and chain-like structures might be an key ingredient for the understanding of the mechanical properties of metallic glasses.

Metallic glasses (MG) are a class of materials consisting of a solid metallic alloy with a lack of long-range order employed in engineering, electronics and as bio-compatible materials^1^,^2^,^3^,^4^. First obtained by Klement *et al*. in 1960^5^, such amorphous metals are obtained by rapid quenching of liquid metals to avoid crystallization and keep the structural disorder. Primordial in many technological applications^3^,^4^, the peculiarities of MGs rely on the short-range and medium-range arrangements of the atoms of the material and thus must be understood at the nanometer-scale. Since the early state of MG research, atomic-scale structural models have been an intense topic of discussion. Following the Bernal’s model, amorphous metals can be pictured as a frozen metallic liquid being a dense arrangement of randomly–packed spheres^6^,^7^. Exemplary for alloys with similar atomic radii, the model does not consider any medium-range or short-range orders however experimentally observed in complex glasses. Further models have proposed a random packing of stereo-chemically defined clusters^8^,^9^, where the bonding between atoms is well-defined and resembles their crystalline forms^10^,^11^. In these models, the long-range order is suppressed in all space directions due to the icosahedral–like symmetry of the clusters^12^,^13^.

Combined theoretical calculations and nano-beam diffraction^14^,^15^ have indeed experimentally observed icosahedral-like diffraction patterns in MG, however always accompanied by large distortions giving rise to face-cubic centered (fcc) and hexagonal closed packed (hcp) configurations. Although these results confirmed the existence of icosahedral clusters, the restriction to the reciprocal space hinders to conclude whether the clusters have complex hybrid hetero-structures^15^ or several metastable structures coexist in the MG as recently suggested^16^,^17^,^18^. With transmission electron microscopy and scanning probe microscopy, such issue can be addressed since the spatial resolution can be brought from hundreds of nanometers down to the atomic scale in the real space. Notably, scanning tunneling microscopy (STM) and atomic force microscopy (AFM) are in principle powerful tools to study two-dimensional amorphous films and reveal their structural characteristics^19^,^20^,^21^,^22^,^23^. Surprisingly, observations of metallic glasses at such atomic-level still remain inconclusive with these techniques^24^,^25^,^26^.

Here, we systematically performed real-space investigations of the Ni_40_Ta_60_ metallic glass surface by means of STM at low temperature in combination with X-Ray Diffraction (XRD) and X-ray Photo-electron Spectroscopy (XPS) to shed light on the atomic-scale structural characteristics of metallic glass.

## Results

**Figure 1:**
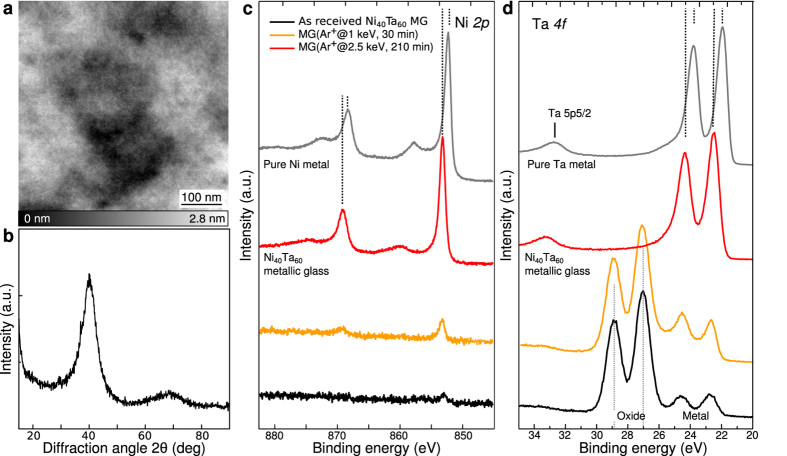
Amorphous nature and bonding character of the Ni_40_Ta_60_ metallic glass. (**a**) Typical large-scale STM overview of the Ni_40_Ta_60_ metallic glass surface, (500 × 500) nm^2^; I = 10 pA, V_*t*_ = 50 mV). (**b**) Typical XRD pattern of the amorphous metal. (**c**) normalized XPS spectra of Ni *2p* and, (**d**) Ta *4f* of the metallic glass with respect to the preparation cycles. The gray curves in both spectra correspond to the pure Ni and Ta metals respectively. As received samples always show a surface Ta-rich oxide layer which can be removed with preparations. After oxide removal, both Ni *2p* and Ta *4f* metal spectra are shifted to higher binding energies compared to their metal analogue as a consequence of their preferential bonding in the metallic glass.

The Ni_40_Ta_60_ metallic glass used in our study were quenched in a home-built apparatus by the crucible free splat-cooling method (10 m.s^−1^) corresponding to a quench rate of ≈10^7^ K/s. Prior to measurements in ultra-high vacuum, the sample was cleaned by a few cycles of sputtering (Ar^+^, 2.5 keV, up to 210 min) and subsequent annealing at 650 K. It is worth mentioning that Ni_40_Ta_60_ is considered as a high temperature MG meaning that its glass temperature *T*_*g*_ is relatively high compared to other metallic glasses (≈1080 K)^27^,^28^. The annealing temperature is 430 K below *T*_*g*_ thus re-crystallization of the glass is not expected. Additionally, all of the samples were found completely amorphous by means of X-ray diffraction and STM overviews after preparations. A typical large-scale constant-current STM image shown in Fig. 1a reveals the lack of long-range order in accordance with the amorphous state of the material, i.e. no crystalline domains, steps and grain boundaries are observed at the surface. Fig. 1b shows a typical XRD pattern of the sample which further confirms the absence of long-range order in the bulk of the material. Therefore, atomic-scale arrangements in whatsoever structures are not spatially extended at both surface and bulk of the material.

### Amorphous nature of the Ni_40_Ta_60_ metallic glass

To understand the bonding character of the glass constituents, we first characterized the surface chemistry by XPS. Wide scan spectra of an as-received sample show a significant oxide peak at the surface that is removed by subsequent preparations (See Supp. Infos). Figure 1c,d show the evolution of the Ni *2p* and Ta *4f* core level spectra with respect to the cleaning procedure. The Ni *2p* peaks (Fig. 1c) are first not visible (black curve) but appear after cleaning (red curve). The Ta *4f* spectra (Fig. 1d) clearly shows an oxidation state 4^+^ for as-received samples (black curve), i.e. Ta_2_O_5_ with a binding energy at 27 eV (Ta *4f7/2*) and 28.8 eV (Ta *4f5/2*), thus we conclude that Ta atoms preferentially segregate at the surface due to the high enthalpy of oxide formation compared to Ni and form a Ta_2_O_5_ adlayer. As-received sample surfaces thus correspond to an amorphous oxide surface layer rather than the *Ni*_40_*Ta*_60_ glass. With sample preparations, the Ta *4f* peak positions shift to lower binding energies at 21.7 eV (Ta *4f7/2*) and 23.6 eV (Ta *4f5/2*) respectively. Although the Ta_2_O_5_ peak positions are in excellent agreement with the literature^29^, the Ta *4f* metallic state spectrum is however shifted by ≈0.5 eV towards higher binding energies relative to the pure metal (gray spectrum). A similar observation is found for the Ni *2p* (dashed lines in Fig. 1c) having ≈1 eV shift towards higher binding energies compared to the Ni pure metal state (gray spectrum). Knowing that the full width of half maximum of each peaks (FWHM_*Ni*_ = 1.16 eV and FWHM_*Ta*_ = 0.94 eV) is comparable with those of the pure metals (FWHM_*Ni mental*_ = 1.19 eV and FWHM_*Ta mental*_ = 0.83 eV), we think that the concomitant shifts of these peaks are due to the preferential bonding between Ni and Ta in the glass. Importantly, the presence of shake-up satellites for Ni *2p* at 862 eV and 877 eV appearing after cleaning is also a clear signature of the metallic nature of Ni-Ta amorphous state. Therefore, both the recovery of the metallic character and the shifts of the peaks to higher bonding energies suggest an important ubiquity of the bonding character of the glass constituent free of contaminants.

### Real-space observations of the Ni_40_Ta_60_ surface by scanning tunneling microscopy

**Figure 2:**
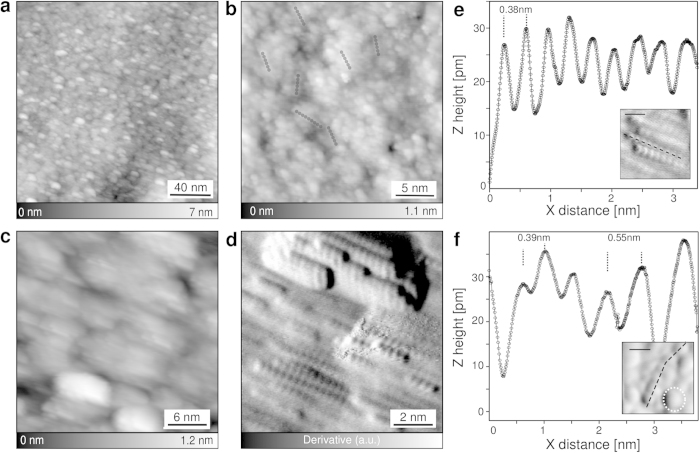
Real-space observation of short- and medium-range order. (**a**) STM overview of the Ni_40_Ta_60_ sample revealing the lack of long-range order, (200 × 200) nm^2^. (**b**) The close-up view of the surface shows that mainly small clusters are observed with few embedded and randomly oriented chain-like structures (dashed lines). (**c**) STM image of an amorphous area composed of small clusters and where a medium-range (MR) order is locally observed. (**d**) Derivative STM image showing that short chain-like structures (≤10 nm) are running parallel favoring the MR order. However, their short lengths, between 2 to 10 nm, avoid arrangements over more than few tens of nms thus keeping the disordered nature of the MG, (I = 10 pA, V_*t*_ = 50 mV). (**e**,**f**) Topographic STM profiles taken along such chain-like structures revealing an atomic periodicity of ≈0.38 nm. The comparison between (**e**) and (**f**) shows that this periodicity locally varies by ≈0.17 nm due to the structural deformability of the chains. Both profiles have been background substracted and taken along the dashed lines of their insets. For clarity, both inset pictures are derivatives of the corresponding STM images, the scale bars are equal to 1 nm.

To reveal the metallic glass structure in the real-space, we systematically performed STM at various sample areas to characterize the typical surface topologies. Figure 2 shows a collection of STM images of the sample surface obtained at 4 K. At scans larger than 50 × 50 nm^2^ and independently on the scanning position, no crystalline structures were observed confirming the lack of long-range order and the absence of re-crystallization after preparations (Fig. 2a). At first glance, close-up STM views (Fig. 2b,c) show that the MG surface consists of small and randomly distributed clusters. Chain-like structures, which the structure varies between 2 to 10 nm, are often found embedded in the field of clusters as marked with dark lines in Fig. 2b. The inset of Fig. 2f is a derivative STM image showing that such chain (dashed line) is surrounded by a few clusters (white dashed circle). The cluster sizes are about 0.6 nm with a sharp distribution around this value (≈0.2 nm). We attribute these clusters to icosahedral-like structures as recently reported in the literature^14^,^15^. Along the chain, the inter-unit periodicity is ≈0.38 nm (profile of Fig. 2e) from the STM data which is much smaller than the cluster size. It thus corresponds to an atomic chain rather than an assembly of icosahedrons^16^. Moreover, the distance between units has an important site-dependent modulation of the bond length (up to ≈150 pm) and the bond angle (≈30°) suggesting the important deformability of the chains. The observation of two embedded structure elements, i.e. icosahedrons and short wires, agrees with the experimental results obtained by nano-beam diffraction reporting mixed fcc and icosahedral structures in metallic glasses and supports recent theoretical works^12^,^14^,^15^,^16^,^17^,^18^. Importantly, both structural motifs must have sizes in the nano-meter range which foster their entanglement and a close-packing. Since there is no long-range order in any directions of the sample, these structures cannot be detected by XRD which is in analogy to amorphous polymers showing strong disorder by XRD pattern although the fundamental molecular motif is well defined.

By symmetry, icosahedral structure avoids any long-range arrangement in contrast to one-dimensional chains. Figure 2c shows that medium-range arrangement of these chains, up to few tens of nanometers, can be sometimes observed at the MG surface. The relative orientation observed in Fig. 2c is induced by the uni-dimensionality of the chain. However, this orientation is strictly random and has no coincidence with the sputtering direction. At the atomic scale (Fig. 2e,f), the regular modulation of 0.38 nm along each chain is clearly resolved confirming their atomic nature. As shown in Fig. 2d and by the profile Fig. 2e, the atomic distance along the chains is *a* ≈ 0.38 ± 0.17 nm whereas the distance between chains is *b* ≈ 0.72 nm. In agreement with the XPS measurements and the measured STM periodicities, we firmly exclude oxidized alloys of Ni and/or Ta as well as pure Ni or pure Ta structures. The measured periodicity along the chain is however in agreement with tetragonal NiTa_3_ structures obtained from crystallographic data.

### Calculated metastable structures of the Ni_40_Ta_60_ metallic glass

**Figure 3:**
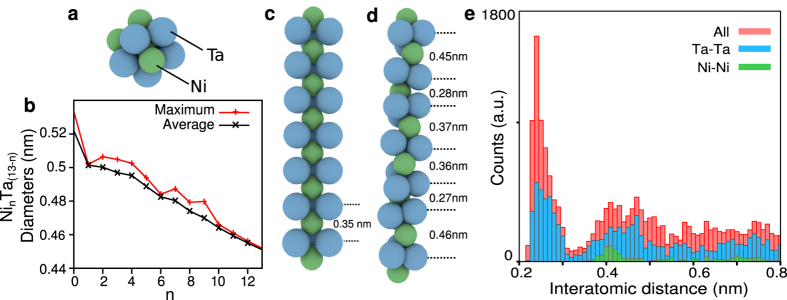
DFT calculations of metastable structures in the Ni_40_Ta_60_ glass. (**a**) Stable Ni_3_Ta_10_ icosahedral structure, Ni and Ta atoms are colored in green and blue respectively. (**b**) Maximum and average diameters of Ni_*n*_Ta_13−*n*_ clusters as a function of their stoichiometry (factor *n*). All Ni_*n*_Ta_13−*n*_ preserve their initial icosahedral geometry after relaxation. (**c**) Stable structure of a linear Ni_8_Ta_21_ chain, the distance between Ta atoms is 0.35 nm in agreement with STM observations. (**d**) Example of a metastable Ni_8_Ta_21_ chain structure after 100 relaxations obtained by minimum hopping method combined with DFT. Among all relaxations, the chains reveal an important structural adaptability. **e**, Histogram of inter-atomic distances for about 100 low energy configurations of Ni_7_Ta_18_ and Ni_8_Ta_21_ chains.

To better understand the observed structural motifs, we performed the minima hopping method coupled to Density Functional Theory (DFT) calculations^30^ (see details in Methods) in order to find the most stable and low energy configurations of both icosahedral and chain-like structures. In agreement with the STM data, we focused on icosahedral clusters (Fig. 3a) and relaxed Ni_7_Ta_18_ and Ni_8_Ta_21_ chains structures (Fig. 3c,d). First, we considered Ni_*n*_Ta_13−*n*_ icosahedral clusters consisting of 13 atoms which have sizes similar to the STM data Fig. 2e. After relaxation, all configurations preserve the initial icosahedral structures as depicted in Fig. 3a. The graph Fig. 3b shows the variation of their diameters depending on their stoichiometry which varies between between 0.44 and 0.54 nm. These values are in good agreement with the STM observations of ≈0.6 nm, which is slightly larger most likely due to tip-sample convolution artifact. Although we cannot conclude to a particular Ni/Ta stoichiometry, the icosahedral geometry is always observed at the surface of the MG. Figure 3c depicts the stable and unrelaxed Ni_8_Ta_21_ chain having a tetragonal structure as obtained by the minima hopping method with BigDFT^31^. The distance between neighboring Ta atoms is 0.38 nm and confirms the inter-unit distance *a* measured between high contrast spots along the chains of the STM image (Fig. 2f). Figure 3d shows a characteristic chain structure obtained after several relaxations using minima hopping method (see Methods). The distance between Ta atoms is marked along the chain and shows important variations. The histograms Fig. 3e shows the Ni-Ni, Ta-Ta atomic distances for about 100 low-energy chains with relaxed and stable configurations respectively. The broad peak at ≈0.4 nm on the blue histogram coincides with the Ta-Ta inter-unit distance along the chain. Its large distribution shows the important variation of the bond lengths between atoms in the chain and reveals that the structure can be strongly deformed while remaining stable, thus favoring a close packed arrangement in the metallic glass.

### Tip-induced relaxations at the metallic glass surface

**Figure 4:**
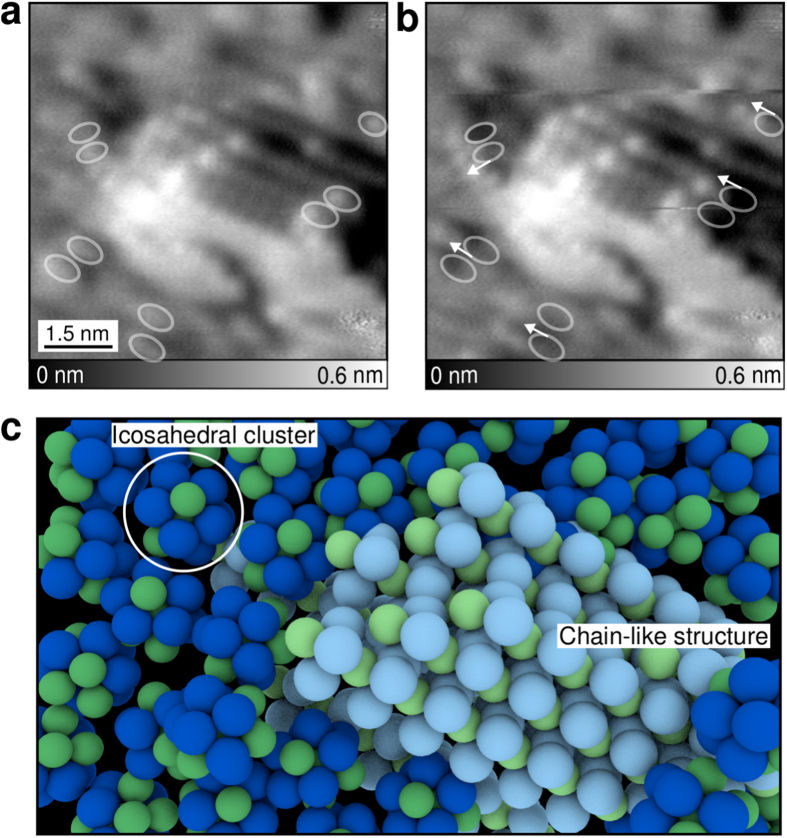
Tip-induced surface relaxations at short-range scale (**a-b**) Constant-current STM image of chains surrounded by icosahedral clusters. While scanning with extremely gentle conditions, numerous tip-induced displacements of clusters depicted by white arrows (see also movie in Supp. Infos.) occur at icosahedron areas while chain-like structure remain stable. The white circles show the initial position of the clusters obtained from (**a**) and moved in (**b**) (I = 10 pA, V_*t*_ = 30 mV). (**c**) Model of the surface morphology: areas of icosahedrons which are weakly bound (dark blue) present high rate of relaxation and might thus act as local shear transition zones. In contrast, chain structures (pale blue) limit these relaxations, Ni and Ta atoms are in green and blue respectively.

To study the interplay between these two metastable structures and the surface dynamics, we investigated the tip-induced relaxation processes obtained with extremely gentle conditions over several STM images while scanning the glass surface. Figure 4 shows two successive STM images extracted from a STM movie (see Supp. Materials). In Fig. 4a, a chain aggregate is atomically resolved and surrounded by icosahedrons. Successive STM images taken at constant-current mode with a tunneling resistance of ≈6 GΩ reveal that numerous relaxation processes occur under the tip action around the chain aggregate. For clarity, we marked few clusters in Fig. 4a with white circles which were moved by the STM tip in the following image (Fig. 4b). The white circles in Fig. 4b show the initial position of these clusters obtained from Fig. 4a and thus reveals the surface dynamics (see also movie in Supp. Infos.). The white arrows (Fig. 4b) point out few of the displacement of clusters which corresponds to a translation mediated by the tip scan. In contrast to the chains remaining immobile during all the STM scans, areas consisting of icosahedrons (dark blue in Fig. 4c) show numerous relaxations of the clusters along few atomic sites. Their preferential motion suggests that icosahedrons are weakly interacting to each other and allows large relaxation processes in contrast to chain area (pale blue Fig. 4c) which are much less sensitive to the tip-induced deformation. We think that these displacement processes can be interpreted as a signature of Johari-Goldstein relaxations (secondary or *β*-relaxation) attributed to translation motion of atoms on a short-range scale^32^,^33^ and appearing well below *T*_*g*_ (here at 4.8 K). Importantly, *β*-relaxation is predicted to be correlated with the volume of shear transformation zones (STZ) of the metallic glass. Because icosahedron areas are weakly interacting and have high *β* relaxation rate, we propose that STZs might locally initiate at these locations where important deformations are allowed. In this picture, incorporating nano-sized chain-like structures in these areas limits these relaxations and thus increases the overall strength of the material^34^,^35^.

## Discussion

Real-space imaging combined with DFT calculations thus demonstrates the coexistence of two well-defined structures in Ni_40_Ta_60_ at the atomic scale: icosahedral clusters and chain-like structures. Consequently, a way to better foresee the MG properties at the macroscale might be to consider them as nano-composites composed of both structures. In this picture, the entanglement of icosahedron areas weakly bond to each other with nano-sized chain-like structures might be a way to define the macroscale properties of the whole glassy material. In close analogy to amorphous polymers, the ratio between these metastable structures, their total sizes and how they interact to each other, thus influencing the Johari-Goldstein relaxation, might be the key ingredients to control the final properties of metallic glasses. Scanning probe microscopy techniques particularly offer the possibility to elucidate the structure and potentially the dynamics of more complex metallic glasses with the prospects of designing tunable amorphous metals with ultra-high glass forming ability and improved physical, chemical and mechanical properties.

## Methods

### Metallic glass synthesis

The Ni_40_Ta_60_ sample was prepared with the crucible free splat-cooling method^27^. A small ball of alloy with a diameter of 3–5 mm is heated inductively in a levitation coil up to its melting point. When this temperature is reached, the RF field is switched off and the sample falls down and is squished between two copper pistons. The resulting splat (d = 25 mm, 60 *μ*m-thick) is cooled down with a rate of ≈10^7^ Ks−1. For the high melting temperatures of Ni_40_Ta_60_ (T_*m*_ = 2300 K) the heating energy was enhanced by applying a CO_2_ laser beam (wavelength: 10.6 *μ*m, 1 kW CW) focused from top to the free levitating sample.

### Spectroscopic experiments

The XPS measurements were performed with a VG ESCALAB 210 spectrometer using monochromatized Al K_*a*_ radiation (1486.6 eV) with an energy resolution better than 0.5 eV for 20 eV pass energy. Normal electron escape angle and a step size of 0.05 eV were used. The binding energy scale was calibrated using a clean gold sample and positioning the Au 4f7/2 line at 84.0 eV binding energy. XRD patterns were recorded using a SIEMENS D500 instrument with monochromatic Cu K*a* radiation (40 kV and 30 mA) at a grazing incidence of 5°.

### Scanning Probe microscopy

The SPM measurements were realized with a low-temperature STM/AFM microscope (Omicron Nanotechnology GmbH) based on a tuning fork sensor (stiffness of k = 1800 N/m, resonance frequency *f*_0_ = 26 kHz, Q factor = 35000) and operated at 5 K in ultrahigh vacuum^36^,^37^. All STM images were recorded in the constant-current mode with the bias voltage applied to the tungsten tip.

### DFT calculations

The configuration space of structures of size 5 ≤ *N* ≤ 30, each having *N* different stoechiometries Ni_*n*_Ta_*N−n*_, were searched for stable, low energy configurations on the density functional theory (DFT) level. To this aim, we employed the minima hopping(MH) method^30^ coupled with the BigDFT^31^. The local density approximation and *U* the HGH pseudopotentials^38^ with 5(10) valence electrons of Ta(Ni) atoms were used. The MH method does a series of successive molecular dynamics and geometry relaxation steps in such a way that the low-energy part of the potential energy surface is sampled sufficiently. To verify the existence and stability of the chains structures, it was enough to continue the MH runs until few elongated structures were collected. Since our aim is finding the geometrical parameter *a*, we ignored the interaction with the substrate in order to reduce the calculation cost.

## Additional Information

**How to cite this article**: Pawlak, R. *et al*. Chain-like structure elements in Ni_40_Ta_60_ metallic glasses observed by scanning tunneling microscopy. *Sci. Rep*. **5**, 13143; doi: 10.1038/srep13143 (2015).

## Supplementary Material

Supplementary Information
