# Supplementary material for: Chain-like structure elements in Ni40Ta60 metallic glasses observed by scanning tunneling microscopy
Source: Sci Rep. 2015 Aug 13;5:13143. doi: 10.1038/srep13143 (PMC4542518; doi:10.1038/srep13143)
Supplement: Supplementary Information [file srep13143-s1.pdf]

Supplementary Information for ”Chain-like structure elements in $Ni_{40}Ta_{60}$ metallic glasses observed by scanning tunneling microscopy”

Rémy Pawlak[1,*], Laurent Marot[1], Ali Sadeghi[1], Shigeki Kawai[1], Thilo Glatzel[1], Peter Reimann[1], Stefan Goedecker[1], Hans-Joachim Güntherodt[1], and Ernst Meyer[1]

[1]Department of Physics, University of Basel, Klingelbergstr. 82, 4056 Basel, Switzerland.
[*]remy.pawlak@unibas.ch

ABSTRACT

This pdf file includes:
- Supplementary Text for Materials and Methods
- Figs. S1 and S2
- Movie of the surface dynamic induced by the STM tip.

Materials and Methods

The metallic glass sample used in our work were synthesized by free splat-cooling method in a home-made apparatus. Fig. S1 describes the working principle of such cooling procedure. A small metallic ball (d = 5 mm) of the desired alloy is heated above its melting temperature and levitates in a water-cooled RF coil. With the help of an additional laser heating, temperatures up to 3800 K can be reached experimentally. When the alloy is melted while being maintained in the coil as shown in the Fig. S1b, the RF is switched off leading to the fall of the melted metal ball within the apparatus. During the fall, the melted metal ball crosses a laser detector aiming to activate the motion of a piston. The two copper part of the piston are rapidly brought into contact (10 $m.s^{-1}$) and trap the metallic ball. This trapping procedure acts as fast-cooling procedure for the metal ball avoiding its crystallization and thus the efficient production of various kinds of metallic glass. During the procedure, the material can be cooled with a rate of $\approx 10^7$ $K.s^{-1}$. The resulting sample is a disk of 25 mm diameter and up to 60 μm thick shown in Fig. S1c.

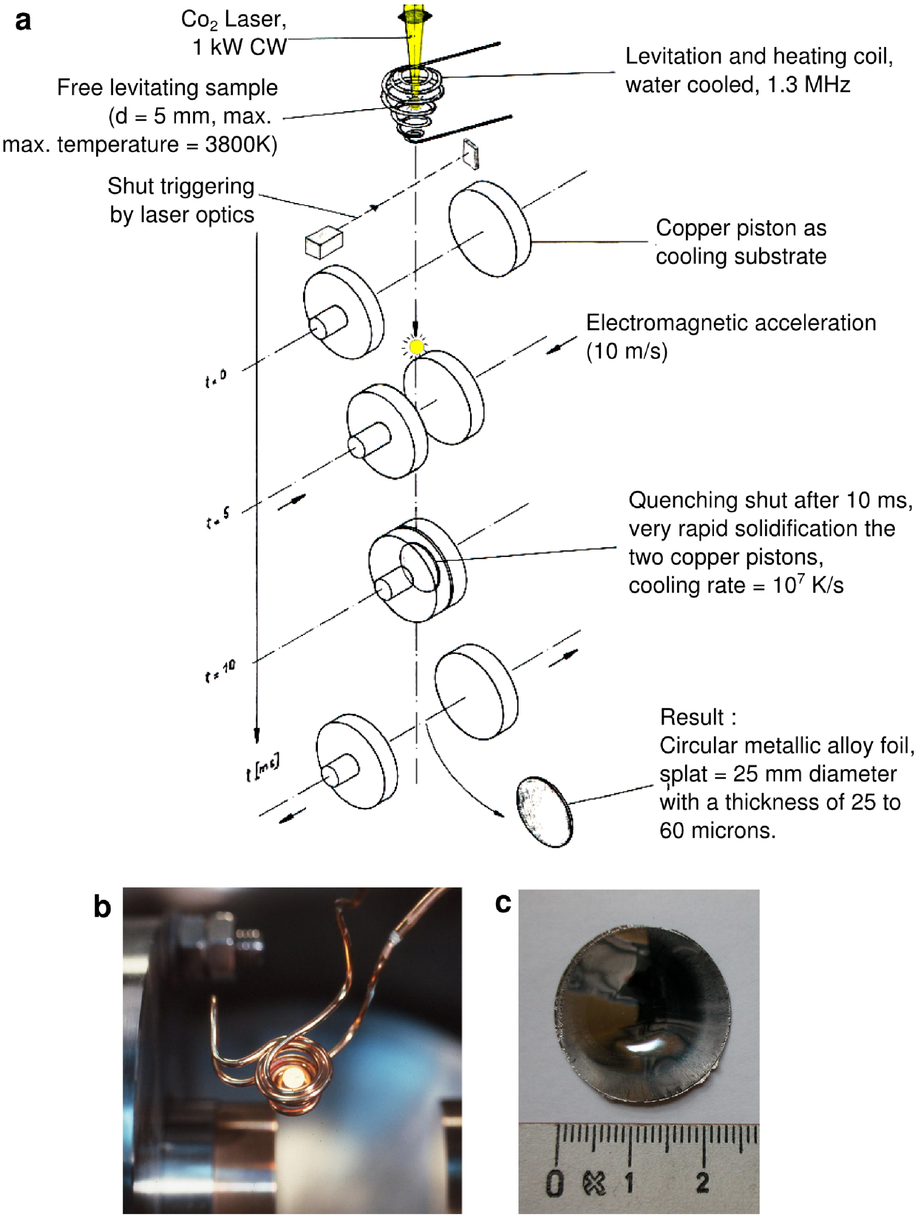

Figure 1. **a**, Schematic of the working principle of the splat-cooling system. The apparatus provides a maximum heating temperature of $\approx$ 3800 K and a cooling rate of 10^7 K.s^{-1} allowing the production of disk of metallic glass. **b**, picture of the levitating material heated above its melting temperature before the cooling procedure and **c** the resulting "splat" after cooling.

XPS characterization of the metallic glass.

Figure S2 shows the evolution of wide scan XPS spectra with respect to the preparation cycles under ultra-high vacuum, consisting of Ar^+ sputtering and annealing at 650 K below the re-crystallization temperature of about 1030 K. As-received samples always show an important oxide layer at the surface of the glass (black curve). During production, the melted alloy is indeed rapidly cooled by being squeezed between two copper plates acting as cooling substrate. Therefore, the large temperature gradient at the interface Cu-metallic glass favors the exchange of materials between the two substrates and explains the traces of Cu on as received samples. The XPS spectra of as-received samples reveal the presence of Cu, O, and C (marked in Fig. S2) at the surface of the glass due to air contamination. The Ni $2p$ peaks are extinguished whereas the Ta $4f$ is uniquely found in its oxide state of Ta_2O_5 (see main manuscript). It is due to the higher chemical reactivity of Ta compared to Ni which promote the segregation of this oxide. By removing the oxide layer with sputtering/annealing, Cu $2p$ peaks are suppressed and the O $1s$ and the C $1s$ vanished in favour of the Ni $2p$ and Ta $4f$ in their metal states. The red curve corresponds to a pure metallic

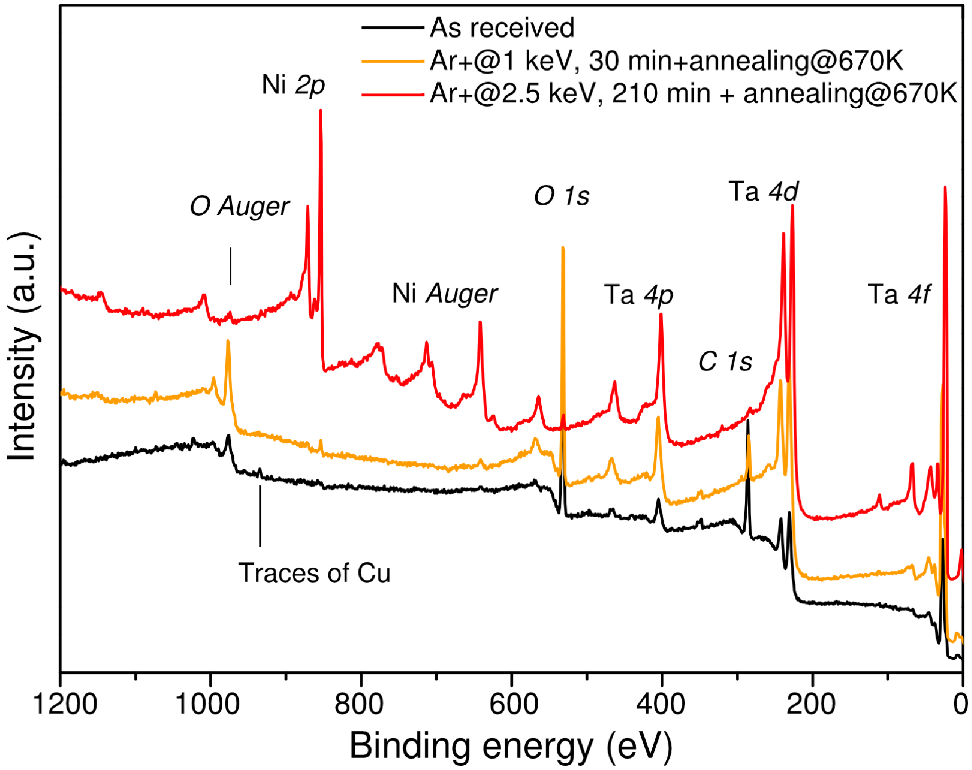

Figure 2. Evolution of the wide-scan XPS spectra of the $Ni_{40}Ta_{60}$ with preparation cycles. The *as received* sample shows an important oxide layer at the surface of the metallic glass which can be removed by few cycles of sputtering and annealing. The XPS spectra of the cleaned samples show the presence of Ni 2*p* and Ta 4*f* peaks in the metal state and do not change with further preparation cycles.

glass obtained after the cleaning process. Although a trace of oxygen is still present, the spectrum does not evolve further with additional preparation cycles. We think that this small oxygen amount is most likely trapped in the glass material during its production conducted in low vacuum. However, we emphasize that the structures observed by STM (icosahedral and chain like) and reported in the main manuscript are not related to oxide structures because their lattice parameters are much smaller than oxidized structures. Rather, oxygen might play a role of defects at the MG bulk and surface. According to this spectrum, the material consists of 29.8 at.% of Ni, 50.9 at.% of Ta, 12.5 at.% of O and 6.8 at.% of C.

Figs. 1c and d of the main manuscript show the XPS spectra evolution with the preparation cycles of the Ni *2p* and Ta *4f*. The red curves correspond to the clean metallic glass NiTa alloy. Full width of half maxima (FWHM) of Ni *2p* and Ta *4f* in the metallic glass and in the pure metals are compared in the following table.

FWHM	Metallic Glass	Pure Metal
Ni	1.16 eV	1.1 eV
Ta	0.94 eV	0.83 eV

Fitting of the core level lines was performed using Doniach-Sunjic (asymmetrical Lorentzian) functions,[1] with a Shirley background substraction,[2] using UNIFIT for Windows (Version 2013) software.[3] A convolution of an asymmetry function, Lorentzian and Gaussian line shapes was used to fit the individual peaks. After this, the intensities were estimated by calculating the integral of each peak; the atomic concentrations were then derived using Scofield sensitivity factors.[4]

The XRD patterns before and after such cleaning treatment do not show any relevant changes of the bulk structure. In other word, the surface of the metallic glass in the oxidized or pure metal states form does not impact the amorphous nature of the bulk. Such observation might be important for future experiments on metallic glasses or for industrial applications.

References

1. S. Doniach and M. Sunjic. J. Phys. C: Solid State Phys., 3:285, 1970.

2. A.D. Shirley. Phys. Rev. B, 5:4709, 1972.

3. R. Hesse et al. J. Anal. Chem., 365:48, 1999.

4. J.H. Scofield. J. Electron Spectrosc. Relat. Phenom., 8:129, 1976
